# A longitudinal study on the effects of psychological stress on proteinuria in childhood steroid-sensitive nephrotic syndrome

**DOI:** 10.1016/j.jpsychores.2019.01.011

**Published:** 2019-06

**Authors:** Lianne Bakkum, Agnes Maresa Willemen, Lydia Zoetebier, Antonia H. Bouts

**Affiliations:** a Department of Public Health and Primary Care, Primary Care Unit, University of Cambridge, Cambridge, United Kingdom; b Department of Clinical Child and Family Studies and Amsterdam Public Health, Vrije Universiteit Amsterdam, Amsterdam, The Netherlands; c Department of Pediatric Nephrology, Emma Children's Hospital, AMC, Amsterdam, The Netherlands; d AMC Medical Research, Amsterdam, The Netherlands.

**Keywords:** Albuminuria, Life experiences, Nephrotic syndrome, Pediatrics, Stress, Psychological, Symptom flare up

## Abstract

**Objective:**

Steroid-sensitive nephrotic syndrome (SSNS) in children is often complicated by one or more relapses, as manifested by the appearance of proteinuria. Besides health-related triggers, psychological stress might be related to relapse. This longitudinal study examined the link between perceived stress, emotional valence (feeling happy vs. unhappy) and daily reported proteinuria, and investigated the temporal relation between stressful events and proteinuria.

**Method:**

Sixteen children (4–13 years) diagnosed with SSNS were included. Patients kept an online diary for an average of 124 days, wherein they reported proteinuria (*n* = 1985 urine samples), perceived stress, emotional valence, medication use and health complaints. Stressful days were determined at the start of the study. Using multilevel analysis, the following associations were tested: (1) the relation between perceived stress, emotional valence and proteinuria, and (2) the temporal relation between stressful days and proteinuria.

**Results:**

Appearance of proteinuria was reported in 410/1985 urine samples. Perceived stress and not emotional valence significantly predicted proteinuria (95% CI [0.11, 0.27]), even five days later. There was a significant temporal association between stressful days and proteinuria (95% CI [0.22, 1.14]). The effect sizes of these associations were small, *f* = 0.04 and *f* = 0.12, respectively.

**Conclusions:**

Our findings suggest that psychological stress may trigger proteinuria in children with SSNS. Future research in larger samples is needed to support our findings.

## Highlights

• Perceived stress was associated with increased proteinuria, up to five days later.
• Emotional valence was not associated with proteinuria.
• Days prior to and after stressful events were associated with increased proteinuria.

## 1 Introduction

Steroid-sensitive idiopathic nephrotic syndrome (SSNS) is a severe kidney disease with proteinuria, hypoproteinemia, hyperlipidemia and the appearance of edema. The etiology of SSNS is not known. SSNS might lead to serious complications, such as infections, venous thromboembolism, and cardiovascular disease [10,12]. Around 80% of SSNS patients show a relapse after the first treatment with corticosteroids. Half of these patients experience frequent relapses (>4 times/year) or become steroid-dependent [31]. Steroid treatment is associated with severe side-effects, e.g., growth retardation, hypertension, osteoporosis and bone fractures [14]. Frequent relapsing patients are often treated with second-line drugs such as cyclosporine, tacrolimus, or mycophenolate mofetil—which may have side-effects, e.g., increased susceptibility to infections, reduced renal function and gastrointestinal complaints [18]. It is known that infections, such as the common cold, are most predictive of relapse [21,34]. Psychological stress has been associated with relapse in other relapsing-remitting diseases, such as inflammatory bowel disease, asthma, multiple sclerosis and psoriasis ([1,6,17,22,26]; [40]). For example, Potagas et al. (2008) found that stressful life events and increased levels of anxiety significantly predicted the risk of relapse in a sample of women with multiple sclerosis. The link between stress and relapse in SSNS has been mentioned by Takahashi et al. [32]. In this study, patients presented more relapses in the period preceding scheduled visits to the outpatient clinic, possibly referring to the role of stress. School events and domestic events also seemed to trigger relapses. With these results in mind, it might be possible that stress triggers relapse in SSNS.

Stress is defined by Grant et al. [13] as “environmental events or chronic conditions that objectively threaten the physical and/or psychological health or well-being of individuals”. As Selye (1956) [41] pointed out, both negative events (distress) and positive events (eustress) may evoke stress. Negative events that may elicit stress in children are, for example, failing a grade in school or the death of a grandparent [7]. Positive events, such as a holiday or a birthday, have also been associated with stress [36], possibly because they impact the daily routine or familiar environment of regular life before and after the stressful event. According to the perseverative cognition hypothesis, stressful events cause people to worry during the period anticipating the stressor, which may affect the neurobiology of stress [5,37]. The degree to which someone appraises an event as stressful, defined as perceived stress, varies among individuals. Both stressful events and perceptions of stress appeared relevant to explain changes in child psychological functioning [39]. Emotional valence (how positive or negative an event is) may also be associated with an individual's response to stress [27]. Based on a stress homeostasis framework, stress is hypothesized to impact biological and psychological functioning ([19]; [41]). According to this framework, negative emotions arise when homeostasis is threatened by potential stressful events [42]. At the same time, positive emotions in reaction to stress can relieve the level of stress and may be a buffer against dysregulation [2]. To summarize, both stressful life events and subjective stress (i.e., perceived stress and emotional valence) may contribute to dysregulated psychological and biological functioning.

It is known that the neuroendocrine system plays a role in the relation between psychological stress and somatic illness. Previous research has established that subjective stress affects physical and/or psychological wellbeing [24,33] and induces changes in the neuroendocrine stress axis, leading to the release of cortisol [35]. Cortisol release due to prolonged stress might trigger negative health effects, such as infections, caused by an under- or overreaction of the body's immune system [6,16,28]. Chen and Miller [6] proposed a model for the role of cortisol in the relation between stress and disease in asthma patients. Their model indicates that stress leads to an increased cortisol release, or a decrease in the sensitivity of cortisol receptors, which in their turn dysregulate the immune system and worsen disease symptoms. A similar process may be underlying relapse in SSNS patients.

**Fig. 1:**
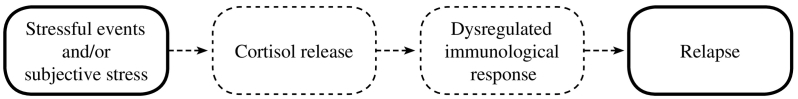
Proposed mechanisms through which stressful life events and/or subjective stress may lead to the occurrence of relapse in SSNS.

Although the exact immunological system underlying SSNS is still unknown, several studies have emphasized an altered immune response, possibly affected by dysregulation of T or B lymphocytes [3,9,23]. Taking into consideration a combination of the exposure to stress and an altered immune response in patients with SSNS, dysregulated cortisol release may lead to an under- or overreaction of the immune system and thus increases the risk of relapse. This may happen due to episodes of prolonged subjective stress (i.e., perceived stress and/or emotional valence), or in the anticipation of stressful events, as a result of worrying. Fig. 1 shows the proposed mechanisms through which stress may affect the likelihood of relapse.

To our knowledge, the study by Takahashi et al. [32] is the only study focusing on the link between stress and relapse in SSNS. However, due to the retrospective character of this study, it is still unknown whether stress precedes relapses. To gain more knowledge about the triggering effect of stress on relapse and the nature of these stressors, it is necessary to test this in a prospective study. In the present study, we prospectively examined the relation between perceived stress, emotional valence and daily reported proteinuria in a sample of children with SSNS. In addition, we investigated the temporal relation between stressful events and proteinuria. We hypothesized that elevated levels of perceived stress and negative emotional valence would be associated with higher proteinuria, one day later. We also expected proteinuria to be higher on days prior to and after stressful events, compared to control days.

## 2 Methods

### 2.1 Study design

A longitudinal research design was used. Patients were followed up to 365 days and used a digital diary to report daily levels of proteinuria, perceived stress and emotional valence. In the original study design, the dependent variable was the number of relapses. However, only five patients relapsed during the study period (*n* = 10). Hence, daily levels of proteinuria were chosen as outcome (*n* = 1985 urine samples). This study controlled for confounding effects of suppressive medication use and health problems as reported by the patients or their parents.

### 2.2 Patients

This study involved 18 children with idiopathic SSNS. Two patients dropped out because of the intensity of the study. Participants consisted of 13 boys and 3 girls, with a mean age of 8.2 years (range 4–13). Inclusion criteria were: age between 5 and 16 years at time of inclusion, SSNS diagnosis, and a relapse rate of at least once per year or a first-time presentation of SSNS. Patients filled in diary entries for in total 1985 days (*M* = 124.07, *SD* = 97.92) days. Proteinuria was reported on all 1985 days. Perceived stress was missing for 6 days (0.5%) and emotional valence for 6 days (0.5%).

### 2.3 Procedure

This study was approved by the Medical Ethical Committee of the Amsterdam university hospital AMC (NL35137.018.11). In six Dutch university hospitals, 48 patients were approached by their nephrologist and received information about the study. Eighteen patients (response rate 38%) and their parents gave written informed consent. Patients were instructed to keep an online diary for 365 days wherein they reported daily proteinuria, perceived stress, emotional valence, changes in medication use and health-related problems. Families received monthly updates on proteinuria, and study progress was reported twice in a newsletter.

### 2.4 Measures

#### 2.4.1 Proteinuria

Proteinuria was measured daily with the Albustix dipstick, which represents a 6-point scale of proteinuria: 1 (*no appearance of albumin*), 2 (*traces of albumin*), 3 (*+*), 4 (*++*), 5 (*+++*), and 6 (*++++*). Values >2 represent the appearance of proteinuria. Relapse indicates a value of 4 (++) and higher on ≥3 consecutive days [31]. Patients were instructed to use the Albustix in the evening, after reporting perceived stress.

#### 2.4.2 Perceived stress

Perceived stress was reported daily on a visual analogue scale in the form of a Distress Thermometer. Patients reported on the question: “Did you feel distressed or nervous today?” using the Distress Thermometer, which produced a score ranging from 0 *(not distressed)* to 100 *(very distressed).* The use of a Distress Thermometer to assess children's psychosocial distress has been validated in pediatric patients with other serious diseases [25,38].

#### 2.4.3 Emotional valence

Emotional valence was reported on the pleasure dimension of the Self-Assessment Manikin (SAM; [4]), a non-verbal pictorial instrument which produced a score ranging from 1 *(very happy, cheerful, or satisfied)* to 9 *(very disappointed, defeated, or sad)*. Bradley and Lang [4] provided data validating the use of the SAM in an undergraduate student sample in measurement of the valence of emotional response.

#### 2.4.4 Stressful days

Four predetermined days were marked as days with stressful events: (1) the children's holiday Saint Nicholas (a Dutch holiday comparable to Christmas) or a school examination, (2) first day of school, (3) birthday, (4) another self-reported stressful day. These events were used in previous research on stress and disease in children [36]. Type of day was a dummy variable. Because this study aimed to investigate the temporal relation between stressful events and proteinuria, seven days before and after a stressful event were marked as stressful days (1), and the seven days after this period were marked as control days (0).

#### 2.4.5 Medication use and health problems

This study controlled for confounding effects of medication use and health problems as reported by the patients themselves or their parents. It is known that corticosteroids and cytotoxic drugs have a suppressive function on the immune system and reduce the relapse rate of NS [10,18]. Infections, such as the common cold, are known to cause relapse [34]. Changes in medication and dosage were reported by the patients' parents. Medication was noted as a dummy variable: days with medication use were marked as 1 and days without medication use were marked as 0. Health problems were also noted as a dummy variable: days with health problems were marked as 1 and days without health problems were marked as 0. All patients' health problems as reported by their parents were included in the study.

### 2.5 Statistical procedure

Multilevel analyses (linear mixed-effects models) were conducted in R (v. 3.5.0) using package lme4 (v. 1.1-17). This is an appropriate type of analysis for study designs with repeated measurements nested within individuals. It takes into account the hierarchical structure of the data, and handles variation in measurement periods and varying numbers of measurements between patients [15,30]. Continuous variables (time, proteinuria, perceived stress and emotional valence) were standardized to a mean of 0 and a standard deviation of 1, to allow for interpretation of the effect sizes of coefficients [20]. The following procedure was used to fit the data into multilevel models. First, an unconditional means model with a random intercept was created, which described the mean value of the outcome variable over time with a zero slope. The independent variables were added step-by-step as fixed effects. Parameter estimates (*β* coefficients) were significant at an alpha level of 0.05. -2 Log Likelihood ratio tests were conducted to test how well the data fit a subsequent growth model. This test uses a χ^2^ – distribution to test differences between the −2 Log Likelihood statistics of two nested models. An alpha level of 0.05 indicated an improved fit of the data to a growth model, given the number of parameters. Effect sizes were determined by computing *f*^2^, which compares the proportion of variance (*R*^2^) explained by two subsequent growth models (one without and one with the effect of interest), and can be interpreted using Cohen's criteria [8]. This approach has been suggested as a suitable method for determining effect sizes in multilevel models ([20]; see also [43]).

#### 2.5.1 The effects of perceived stress and emotional valence on proteinuria

First, a time variable was created to represent the change in proteinuria for each patient. Per patient, each diary entry was given a number. Multilevel analyses were conducted with proteinuria as outcome, and time, medication use, health problems, perceived stress and emotional valence as predictors. Further, analyses were conducted with proteinuria leaded with one day and lagged with seven days, to explore the effect of perceived stress on proteinuria for the previous day and subsequent days.

#### 2.5.2 Validation of stressful days

To test the validity of the stressful events, multilevel analyses were conducted to examine whether patients reported higher levels of perceived stress and negative emotional valence on stressful days vs. control days. Medication use, health problems, and type of day were added to models with perceived stress and emotional valence as the outcome.

#### 2.5.3 The effect of stressful days on proteinuria

To investigate the temporal relationship between stressful days and proteinuria, multilevel analyses were conducted with proteinuria as outcome, and medication use, health problems, and type of day as predictors.

## 3 Results

### 3.1 Descriptive statistics

Together, the 16 participants in this study filled in a total of 1985 diary entries. Patient characteristics are presented in Table 1. In total, fifteen days were determined as days with stressful events. Appearance of proteinuria was reported on 410/1985 days. Medication use was reported on 1399/1985 days (17% missing). Health complaints were reported on 441/1985 days, and mainly included having the common cold (94%). Other reported health issues included allergic reactions, vomiting, diarrhea, fever, stomach ache, headache, sore throat and earache. Multilevel analyses showed that patients who relapsed (*n* = 5) did not show different levels of perceived stress than patients who did not relapse (*p* = .437), nor did they show different levels of emotional valence (*p* = .116). Table 2 presents descriptive statistics of the study variables.

**Table 1:** Patient characteristics (*N* = 16).

Subject	Gender	Age	Duration of study participation	Diary entries	Adherence	Relapses
			*n (days)*	*n*		*n*
1	Male	10	435	193	44%	–
2	Male	7	410	99	24%	–
3	Male	13	387	55	14%	1
4	Male	9	20	9	45%	–
5	--	--	--	--	--	--
6	Male	9	456	283	62%	4
7	Male	5	526	26	5%	–
8	Male	9	417	372	89%	2
9	--	--	--	--	--	--
10	Female	9	415	274	66%	2
11	Male	6	412	246	60%	–
12	Male	7	397	135	35%	–
13	Male	11	170	62	36%	–
14	Male	5	24	19	79%	–
15	Male	9	202	28	14%	–
16	Male	8	202	28	14%	–
17	Female	4	174	127	73%	1
18	Female	10	112	29	26%	–
Totals			*n* = 4759	*n* = 1985	42%	*n* = 10

*Note.* The number of diary entries equals the number of daily proteinuria reports. Relapse: ≥3 consecutive days an Albustix value of 4 (++) or higher. Adherence rate: percentage of diary entries during study participation (diary entries/days of study participation). Patients 5 and 9 dropped out of the study.

**Table 2:** Descriptive statistics.

	*n*	*M*	*SD*	Range
Proteinuria	1985	1.35	0.83	1–6
Stressful days	106	1.67	1.36	1–6
Control days	45	1.29	0.51	1–3
Perceived stress	1985	23.11	24.95	0–100
Stressful days	106	28.73	26.16	0–100
Control days	45	25.02	26.77	0–75
Emotional valence	1985	2.95	1.27	1–9
Stressful days	106	2.80	1.08	1–6
Control days	45	2.91	1.06	1–5

*Note.* Seven days before and after the stressful event (*n* = 15 events in total) were marked as stressful days. The seven days after this period were marked as control days. Perceived stress was reported on the Distress Thermometer. Emotional valence was reported on the SAM [4].

### 3.2 The effects of health problems, medication and age

Table 3 shows a summary of the multilevel model. Adding time did not improve the model fit (Model 1). Health problems and medication improved the model fit (Model 2). As expected, proteinuria was significantly higher on days when children reported health problems (*M* = 1.43, *SE* = 0.10, 95% CI [1.21, 1.64]) than on days without health problems (*M* = 1.10, *SE* = 0.09, 95% CI [0.90, 1.3], *p* < .001). Similarly, proteinuria was significantly higher on days when patients used medication (*M* = 1.39, *SE* = 0.09, 95% CI [1.19, 1.59]) than on days when they did not (*M* = 1.14, *SE* = 0.11, 95% CI [0.92, 1.36], *p* = .002). Controlled for medication and health problems, age was significantly associated with proteinuria (*β* = −0.16, *SE* = 0.07, 95% CI [−0.29, −0.03], *p* = .045). Because age was not significantly associated with either perceived stress (*β* = 0.18, *SE* = 0.13, 95% CI [−0.08, 0.44], *p* = .203) or emotional valence (*β* = 0.16, *SE* = 0.14, 95% CI [−0.11, 0.44], *p* = .270), age was not included as a covariate in subsequent analyses.

**Table 3:** Summary of the multilevel model for the effects of perceived stress and emotional valence on proteinuria.

	Unconditional means	Model 1	Model 2	Model 3	Model 4
Parameters	*β (SE)*	*β (SE)*	*β (SE)*	*β (SE)*	*β (SE)*
Fixed effects					
Intercept	−0.03 (0.10)	0.08 (0.10)	−0.41 (0.13)	−0.45 (0.15)	−0.44 (0.15)
Time		−0.11 (0.03)***	−0.17 (0.03)***	−0.16 (0.03)***	−0.16 (0.03)***
Health problems			0.39 (0.07)***	0.45 (0.07)***	0.45 (0.07)***
Medication use			0.30 (0.09)**	0.34 (0.10)***	0.34 (0.10)***
Perceived stress				0.19 (0.04)***	0.17 (0.04)***
Emotional valence					0.06 (0.03)*

Model summary					
−2 Log Likelihood	5462	5459	4787***	4767***	4769
No. of estimated parameters	2	4	6	7	8

*Note. β* = parameter estimate in the multilevel model, *SE* = standard error, −2 Log Likelihood = deviance statistic. Dichotomous variables (health problems and medication use) are marked as 0–1.

⁎ *p* < .05.

⁎⁎ *p* < .01.

⁎⁎⁎ *p* < .001.

### 3.3 The effects of perceived stress and emotional valence on proteinuria

Perceived stress (Model 3) further improved the model fit (−2 Log Likelihood = 4767, Δχ^2^(Δ*df* = 1) = 20, *p* < .001). Perceived stress had a significant positive effect on proteinuria on the same day (*β* = 0.19, *SE* = 0.04, 95% CI [0.11, 0.27], *p* < .001), one day later (*β* = 0.19, *SE* = 0.04, 95% CI [0.10, 0.27], *p* < .001), but also on the previous day (*β* = 0.12, *SE* = 0.04, 95% CI [0.04, 0.20], *p* = .003). Adding emotional valence did not improve the model fit. As shown in Table 4, perceived stress was significantly associated with proteinuria up to five days later. The effect size of perceived stress on proteinuria on the same day was *f*^2^ = 0.04, which is considered a small effect [8].

**Table 4:** Fixed effects results of the multilevel models for the effect of perceived stress on proteinuria.

Proteinuria	*β*	*SE*	95% CI
Same day	0.19⁎⁎	0.04	[0.11, 0.27]
1 day later	0.19⁎⁎	0.04	[0.10, 0.27]
2 days later	0.13⁎	0.04	[0.04, 0.21]
3 days later	0.14⁎⁎	0.04	[0.05, 0.22]
4 days later	0.13⁎	0.04	[0.04, 0.21]
5 days later	0.12⁎	0.04	[0.03, 0.20]
6 days later	0.05	0.04	[−0.03, 0.14]
7 days later	0.05	0.04	[−0.04, 0.13]

*Note.* Daily reported perceived stress = independent variable, daily reported proteinuria = dependent variable. *β* = parameter estimate of perceived stress in the multilevel model, *SE* = standard error. Proteinuria was measured with the Albustix dipstick. Perceived stress was reported on the Distress Thermometer. Analyses were controlled for the effects of health problems and medication use.

⁎ *p* < .01.

⁎⁎ *p* < .001.

### 3.4 The effect of stressful days on proteinuria

Type of day had a significant effect on daily reported perceived stress (−2 Log Likelihood = 345, Δχ^2^(Δ*df* = 4) = 3902, *p* < .001). Levels of perceived stress were significantly higher on stressful days (*M* = 29.90, *SE* = 8.23, 95% CI [11.30, 48.49], compared to control days (*M* = 20.61, *SE* = 8.49, 95% CI [1.75, 37.47], *p* = .003). There was no association between stressful days and emotional valence. Table 5 shows a summary of the multilevel model for the effect of stressful days on proteinuria. Type of day (Model 3) significantly improved the model fit (−2 Log Likelihood = 383, Δχ^2^(Δ*df* = 1) = 4404, *p* < .001). Stressful days were significantly associated with elevated proteinuria (*β* = 0.68, *SE* = 0.24, 95% CI [0.22, 1.14], *p* = .005). Proteinuria was higher on stressful days (*M* = 1.47, *SE* = 0.41, 95% CI [0.46, 2.49]) compared to control days (*M* = 0.90, *SE* = 0.43, 95% CI [−0.11, 1.93], *p* = .005). The effect size was *f*^2^ = 0.12, which is considered a small to medium effect [8].

**Table 5:** Summary of the multilevel model for the effect of stressful days on proteinuria.

	Unconditional means	Model 1	Model 2	Model 3
Parameters	*β (SE)*	*β (SE)*	*β (SE)*	*β (SE)*
*Fixed effects*				
Intercept	−0.03 (0.10)	0.08 (0.10)	−0.41 (0.13)	−0.83 (0.86)
Time		−0.11 (0.03)⁎⁎⁎	−0.17 (0.03)⁎⁎⁎	−0.36 (0.15)⁎
Health problems			0.39 (0.07)⁎⁎⁎	0.32 (0.34)
Medication use			0.30 (0.09)⁎⁎	0.47 (0.97)
Stressful days				0.68 (0.24)⁎⁎

*Model summary*				
−2 Log Likelihood	5462	5459	4787⁎⁎⁎	383⁎⁎⁎
No. of estimated parameters	2	4	6	7

*Note. β* = parameter estimate in the multilevel model, *SE* = standard error, −2 Log Likelihood = deviance statistic. The reference category for stressful days is control day. Dichotomous variables (health problems, medication use, and stressful days) are marked as 0–1.

⁎ *p* < .05.

⁎⁎ *p* < .01.

⁎⁎⁎ *p* < .001.

## 4 Discussion

This study addressed the relation between stress and proteinuria in a sample of children with SSNS. Because individuals differ in the degree to which they appraise an event as stressful, this study investigated both the effects of subjective stress (perceived stress and emotional valence) and stressful events. It was hypothesized that elevated levels of perceived stress and negative emotional valence were associated with higher proteinuria. Also, it was hypothesized that proteinuria was higher on days prior to stressful events, compared to control days. Our results show that perceived stress was associated with the appearance of proteinuria on the same day, up to five days later, but also on one day earlier. Further, the results indicate higher proteinuria prior to and after days with stressful events.

Corresponding with our hypothesis, daily levels of perceived stress were associated with higher proteinuria, accounting for the influence of suppressive medication and reported health complaints. Emotional valence was not associated with proteinuria. As expected, children showed higher proteinuria on days when they felt more distressed or nervous, and also on one day later. Somewhat surprisingly, this effect was also found for one day earlier, although smaller in magnitude. This might reflect the usual effect that feelings of distress gradually increase instead of suddenly peak for a day [35]. After controlling for emotional valence, perceived stress remained a robust predictor of proteinuria. This suggests that perceived stress, and not positive vs. negative mood state, might predict proteinuria. The results on perceived stress seem consistent with previous research in other relapsing-remitting diseases (e.g., [17]). The absence of an effect of emotional valence might be due the low variance of the scores. On most days, children reported towards the lower end of the scale (feeling rather happy instead of unhappy). Another reason might be that emotional valence interacts with distress, resulting in a multiplicative effect, as suggested by Russell [27]. Because of power limitations, this interaction could not be reliably tested in the current study.

The days prior to and after stressful events were associated with higher proteinuria. These results are in line with those from earlier studies on stressful events and relapse in other relapsing-remitting diseases [1,6,17,22,26], and might be explained by the effect of worrying during the period before the stressful event. Previous study results have shown that the intensity of worrying mediated the relation between stressful events and reported somatic complaints [37].

### 4.1 Limitations

Because of the sample size of the study, our results need to be interpreted with caution. Temporary elevated levels of proteinuria do not always result in relapses. Nevertheless, our findings indicate a moderate effect of stressful days on proteinuria. Another limitation is the large variation in diary entries. Because of the intensity of the study, the adherence rate was rather low (42%) and two patients dropped out of the study. This resulted in a limited number of data points. Further, the relatively low response rate, the overrepresentation of boys (13/16 patients) and the underrepresentation of adolescents limit the generalizability of our findings. Another limitation is the age range of our sample (4–13), with only four children aged above 10 years old. Future research should examine the effects of stress on relapse in SSNS in larger samples.

### 4.2 Implications

This is the first longitudinal study that has attempted to examine the effects of stress on proteinuria in patients with SSNS. The combination of our findings on perceived stress and stressful events, while preliminary, suggests a temporal relation between stress and proteinuria. This study may imply that the reoccurrence of relapses might be limited by reducing stress in children with NS. Therefore, in the treatment of NS, attention should be paid to children's subjective stress experience and the anticipation of stressful situations, for example, by systematically monitoring stress [44] or by offering interventions focused on children's coping with stress [45]. This study extends the findings by Takahashi et al. [32], who retrospectively addressed the link between stress and relapse in SSNS, and adds to the broader literature on stress and disease outcomes [6,16,28]. As mentioned previously, the immunological mechanism of the relation between stress and proteinuria is still unknown. This is an important issue for future research.

## Sources of support

This research was funded by the Dutch Kidney Foundation, The Netherlands [SB191]. Lianne Bakkum is supported by the Wellcome Trust [208155/Z/17/Z].
